# Take a load off: skeletal implications of sedentism in the feet of modern body donors

**DOI:** 10.1093/emph/eoad041

**Published:** 2023-12-23

**Authors:** Malorie E Albee

**Affiliations:** Department of Sociology and Anthropology, Northern Michigan University, Marquette, MI, USA

**Keywords:** osteoarthritis, entheseal changes, sedentism, tarsals, metatarsals

## Abstract

**Background and Objectives:**

Modern biocultural environments continue to place selective pressures on our skeletons. In the past century, a major cultural pressure has been the rise in sedentism. However, studies considering the effects of sedentism on the foot have largely considered pathological changes to the gross foot without particular regard for the pedal skeleton. To address this gap in the literature, temporal trends in the development of osteoarthritis and entheseal changes on the tarsals and metatarsals were analyzed in the context of biodemographic data for recent modern humans.

**Methodology:**

The sample utilized for this project is comprised of 71 individuals from the William M. Bass Donated Skeletal Collection, with birth years ranging from 1909 to 1993. Temporal trends in osteoarthritis and entheseal changes were determined via ANCOVA, using year of birth as the explanatory variable and biodemographic variables (age, sex, stature, body mass index and tibial robusticity) as covariates.

**Results:**

Results indicate that entheseal changes and osteoarthritis have decreased over time, and these trends are statistically significant. Temporal trends in pedal entheseal changes and osteoarthritis vary by sex.

**Conclusions and Implications:**

The increase in sedentary behavior over time has usually been framed as a net negative for human health and well-being. However, considered in isolation, the decrease in entheseal changes and osteoarthritis presented here might be considered a positive development as they suggest overall less stress on the modern human foot. This study also has the potential to inform the health sciences and general public about biocultural contributors to modern foot health.

## INTRODUCTION

The increase in sedentary behavior over time has typically been framed as a net negative for human health and well-being [1–4], especially given our evolutionary past as highly active hunters and gatherers. For example, in their 2020 review of health risks related to sedentism, Park *et al*. [2] report that high levels of sedentary behavior are associated with increased morbidity and mortality, including cardiovascular diseases, metabolic diseases (diabetes mellitus, hypertension, dyslipidemia and obesity), cancer, osteoporosis, chronic knee pain, depression and cognitive function. These negative effects may result from a mismatch between the environments that shaped the evolution of the bipedal human body and the environments that modern humans now inhabit [5–7]. Specifically, modern humans in Western, industrialized nations are often significantly less physically active than our hunter-gatherer ancestors. Even when engaging in sedentary behavior, modern hunter-gatherers sustain low levels of muscle activity by squatting rather than sitting [8]. This is not the case for time spent inactive while sitting in chairs; therefore, there is an evolutionary mismatch even in our inactivity [8]. Many bioarchaeological and paleoanthropological studies have linked sedentism with increased gracility of the skeleton, especially over the Neolithic Demographic Transition when humans became more sedentary [9–12]. However, additional studies on the effects of sedentism on skeletal health have yet to be conducted.

In addition to the negative effects of sedentism on heart health, obesity and overall quality of life during the last 100 years, this transition also potentially included positive consequences for the feet. The health of feet can significantly impact overall body health, mobility and quality of life because they bear the weight of the body. For example, Kerrigan *et al*. [13] found that running shoes were associated with increased joint torques throughout the lower limb joints (hip, knee and ankle) as compared to running barefoot. This minor change to the foot can cause issues throughout the body. The American Podiatric Medical Association solicited public opinions on foot heath and found that not only did 77% of US adults experience foot problems, but also that regular foot pain was significantly associated with health problems elsewhere in the body [14]. These other problems include weight issues, back pain and discomfort, joint pain and arthritis, knee pain and discomfort and even heart issues, which restricted their ability to be physically active [14]. It is possible that many of these issues are related to sedentary behavior, although this is not explicitly identified as such by APMA. Foot health is therefore a key component in understanding overall quality of life and the effects of sedentism on the modern human body. In this study, I aim to document the effects of sedentism on the foot skeleton that are accumulated throughout the life course.

### Secular trends in sedentism in the USA

Sedentary behavior is defined as sitting or reclining behavior that expends ≤ 1.5 METs (metabolic equivalents) while awake [15]. In the USA, it is estimated that the average American (aged ≥ 6 years) spends approximately 7.7 of their waking hours in sedentary behaviors [16, 17], though a more recent study found that adults aged 45 years or over spend as many as 12.3 hours of the waking day in sedentary behaviors [18]. When not engaging in sedentary behaviors, many Americans only engage in light physical activity (1.6 ≤ MET < 3.0), not venturing into the moderate-to-vigorous intensities (3.0+ MET) that are preferred for overall health [19]. In general, then, the average American does not spend much of the day in active standing, walking, or running.

People have become more sedentary over time, particularly in the last 50 years [20]. For example, Ng and Popkin [21] estimate that the amount of time spent in sedentary behaviors increased by approximately 40% from 1965 to 2009. Du *et al*. [22] report that the proportion of American adults not meeting national guidelines for physical activity (150 minutes per week of moderate-to-vigorous activity) and spending greater than 6 hours per day in sedentary behaviors has increased from 16.1% in 2007–2008 to 18.8% in 2015–2016. These trends can be attributed to changes in occupational physical activity as well as decreased physical activity outside of work.

Occupations requiring moderate-to-vigorous physical labor have largely been replaced by ‘cognitive work’ that relies on technology [1]. In 1950, there were more people in high-activity occupations than low-activity occupations, while in 2000, twice as many people held low-activity occupations than high-activity occupations [20]. Tudor-Locke*et al*. [23] estimate that in 2003, 78–88% of American workers held sedentary or low-activity occupations. In 2016, the Pew Research Center reported that employment in cognitive work occupations increased by 77% from 1980 to 2015 [24]. These occupations increasingly rely on intense technology use instead of physical labor [25] and contribute to rises in sedentary behavior.

Additional major drivers of time spent sedentary include the increase in the availability and affordability of indoor entertainment and air conditioners [20, 26] which made staying inside attractive and comfortable for both adults and children. Coupled with this shift to indoor entertainment is a trend among North Americans in general, but women in particular, spending less time in physical activity in the home undertaking domestic tasks like cooking and cleaning [20, 21]. These have likely decreased because of women’s increased participation in the workforce as well as the ease of acquiring processed food and increasing technologies that can automate domestic activities [20, 21]. Finally, suburbanization and ‘urban’ sprawl have contributed to the average North American spending more time in their cars than ever before [26]. In their daily lives both during and outside of work, Americans’ sedentary behavior has increased over the past 100 years.

### Effects of sedentism on the foot

There are few studies that consider the direct effects of sedentism on foot health. Studies attempting to make these direct connections have found statistically significant relationships between sedentary lifestyles and the development of plantar fasciitis [27] and plantar fascial heel pain [28], and additionally found that having a sedentary lifestyle is the most significant independent effect on plantar fasciitis [27]. Other studies link physical inactivity, especially in the workplace, to increased tibio-tarsal breadth [29], decreased blood flow in the foot [30], decreased mean arterial blood pressure in the ankle [30], significant foot swelling [31–34], decrease in temperature of the foot skin and foot muscles [31, 33] and an increased risk of developing plantar ulcers for diabetic individuals [35, 36]. The majority of the effects of sedentism identified in these studies are temporary, and the authors often indicate that the negative effects may be reversed through an increase in physical activity and a reduction in time spent in sedentary behaviors [30–36]. There is, therefore, a lack of studies concerning long-term or permanent effects of sedentism on foot health, particularly of the pedal skeleton.

One way to assess long-term effects of sedentism on the foot skeleton is to consider temporal trends in entheseal changes and osteoarthritis in the foot. Entheseal changes are a skeletal indicator of the attachment site of a muscle, tendon or ligament to bone [37]. While their etiology is unclear, they are thought to form through stress of the insertion site, which increases blood flow and stimulates bone formation processes [38]. This process is not always pathological and instead may be a result of the differences in elastic moduli between bone and connective tissue [39]. Therefore, muscle use should be expected to initiate bony changes at the tendon insertion site throughout life as a ‘normal’ response to mechanical loading [40]. Studies have linked Achilles (calcaneal) tendon and plantar calcaneus entheseal changes with physical activity [41] and occupation [42]. Specifically, Godde and Wilson Taylor [41] found that the calcaneal tendon showed more robust entheseal changes in more physically active individuals compared with those who are less physically active. Similarly, studies comparing entheseal changes among occupations report that there are statistically significant differences in entheseal changes of the calcaneal tendon between Italian farmers and merchants of the late 19th and early 20th centuries [42]. These entheseal changes are more robust in the farmers, and the authors attribute this to higher levels of mechanical loading of the limbs in farmers as compared to other occupations [42]. Entheseal changes of the *Adductor hallucis* m. on the first proximal phalanx have also been related to locomoting over hard substrates [43]. Many other insertion sites of the foot have not been explored, though whether this is due to their infrequent presence or research oversight is unknown. Given the positive relationships between entheseal changes and physical activity seen elsewhere in the skeleton [41, 42, 44–50], this line of evidence has potential to be applicable to understanding changes in the feet as well. Connections between sedentary behavior and entheseal changes in the foot have not yet been explored.

Osteoarthritis, or degenerative joint disease, is ‘a multifactorial degenerative disorder involving focal, progressive loss of articular (hyaline) cartilage, often accompanied by marginal (osteophyte) lipping and articular surface deterioration from direct bone-on-bone contact’ [9]. Osteoarthritis has long been used as a proxy for increased physical activity, although its etiology is complex [51, 52]. Generally, an increased presence or severity of osteoarthritis is often interpreted as evidence of increased levels of physical activity. Evidence of foot osteoarthritis being linked to activity patterns can be found in studies examining osteoarthritis in relation to occupation. For example, Armenis *et al*. [53] compared foot and ankle osteoarthritis in former professional soccer players to the general populace and found that the soccer players had more signs of cartilage degeneration than the control group. Statistically significant differences in foot osteoarthritis between individuals of differing socioeconomic status and occupation have also been found [54]. In this study by Roddy *et al*. [54], individuals in managerial and professional occupations exhibited the lowest levels of foot osteoarthritis while individuals in routine and manual occupations exhibited the highest levels of foot osteoarthritis. The authors of this study do not provide an explanation for these findings, but it is probable that they are related to differing levels of occupational physical activity.

Bioarchaeologically, foot osteoarthritis is more frequent in males associated with horseback riding [55, 56]. Foot osteoarthritis has been found to decrease with the transition to agriculture (and increased sedentism) in North America and increase following European contact [9, 57]. Larsen [9] suggests that the latter pattern is related to the practice of Native American males being drafted into a mission labor system that involved walking long distances while carrying heavy loads. Overall, foot osteoarthritis is often not reported in bioarchaeological studies. However, considering the weight-bearing and locomotive functions of the foot bones, foot osteoarthritis has the potential to be particularly valuable in understanding the effects of physical activity on the skeleton.

### Study objectives and hypotheses

Although the cultural environment of the foot has changed considerably over the last 100 years in the USA, the effects of these recent trends on the pedal (foot) skeleton have not been documented. Documenting these trends can contribute to our understanding of how the foot skeleton embodies nationwide economic and cultural changes that occur during the life course. Therefore, this article aims to answer the following question: To what degree has sedentism impacted pedal health over the last 100 years in the USA?

Considering that the amount of time that Americans spend in sedentary behaviors has increased in the past 100 years, this study tests the hypothesis that pedal entheseal changes and osteoarthritis are negatively associated with year of birth (Hypothesis 1); that is, individuals born later have less severe entheseal changes and osteoarthritis than those born earlier. If loading on the foot has been reduced, pedal entheseal changes and osteoarthritis should decrease accordingly. This hypothesis is supported by previous studies that have identified decreases in foot osteoarthritis related to increased sedentism with the advent and intensification of agriculture [9, 57]. This study also tests the hypothesis that if entheseal changes and osteoarthritis are negatively associated with year of birth, the magnitude of these differences will vary according to sex (Hypothesis 2). This hypothesis is supported by studies like Donaldson, Montoye, Imboden, and Kaminsky [58], which assessed data from participants in research studies at Ball State University’s Clinical Exercise Physiology Laboratory between 2006 and 2014 and found that women spent less time in sedentary behavior than men, and had fewer breaks in sedentary behavior, especially on Saturdays and Sundays. On weekdays, men tended to spend less time in sedentary behaviors and take fewer breaks [58]. These authors, and others, suggest that this is related to women performing housework, childcare, yardwork and other household chores on the weekends [58, 59]. Together, these studies suggest men and women engage in differing levels of sedentary behavior and could therefore be expected to show varying relationships between entheseal changes and osteoarthritis and year of birth.

By identifying temporal trends in pedal health in a historic, industrial, and habitually shod population, this research contributes to the body of literature characterizing the health of the modern human foot and embodiment in the skeleton. It could indicate that the foot in particular and the skeleton in general is capable of responding to cultural shifts in 100 years and potentially provides bioarchaeologists with another avenue of inquiry for understanding cultural practices and their physical effects in the past. This study utilizing a known donated skeletal collection will be informative to future bioarchaeological research in which less biodemographic information is available. By identifying the effects of sedentary behavior on the expression of entheseal changes and osteoarthritis, this study can provide further avenues of paleopathological research to identify the effects of cultural practices on the foot skeleton in the past. Human evolutionary history, especially following the advent of agriculture, is characterized by increasing sedentism over time, and this trend has been extended and intensified in recent years. How has this affected foot skeletal health?

## MATERIALS AND METHODS

### Sample

The sample consists of 71 adults (28 women, 43 men) from the Bass Donated Skeletal Collection at the University of Tennessee-Knoxville Forensic Anthropology Center. These are all white individuals with birth years ranging from 1909 to 1993 and provided ages at death ranging from 19 to 93 years old (Table 1). The majority of individuals in the collection are from Tennessee and the Southeastern United States. This homogeneity in region and ancestry is an advantage in this study as it reduces confounding variables. The Bass Collection was selected for this research due to its high preservation and contextual information as well as the ethical policies under which it was created.

**Table 1.** The total sample utilized in this research, divided by year of birth, age at death, and sex categorizations

	Born 1900–1925		Born 1926–1950		Born 1951–1975		Born 1976–2000		
	Female	Male	Female	Male	Female	Male	Female	Male	Total
18–25 years	0	0	0	0	0	0	2	1	3
26–35 years	0	0	0	0	0	2	0	2	4
36–45 years	0	0	0	0	3	5	0	2	10
46–55 years	0	0	0	0	2	15	0	0	17
56–65 years	0	0	4	4	1	3	0	0	12
66–75 years	0	0	9	5	0	0	0	0	14
76–85 years	0	0	2	1	0	0	0	0	3
86 + years	4	3	1	0	0	0	0	0	8
Total	4	3	16	10	6	25	2	5	71

One foot was examined for each individual. This was usually the right foot, except in five instances in which one or more of the bones of interest was missing or damaged on the right side. In this case, the left foot was used. It is assumed that the feet are similar within an individual, allowing right and left sides to be used interchangeably. This is reasonable because prior studies have found the talus and calcaneus to be essentially symmetric [60, 61]. Individuals with noticeably different bone sizes or pathological expressions between sides were excluded from this study.

### Methods of data collection

To test the proposed hypotheses, this project investigates osteoarthritis and entheseal changes in the tarsals and metatarsals. Typically, entheseal changes have been characterized as ‘normal’ responses to physical activity while osteoarthritis is characterized as a degenerative response to physical activity. This is likely the case because entheseal changes are not as frequently reported to cause pain or physical impairment as is common for osteoarthritis. Therefore, by considering them together, this study includes assessment of both ‘healthy’ and ‘unhealthy’ responses to the physical and cultural environment that might accumulate over time.

#### Entheseal changes

Entheseal changes were recorded for the calcaneus, the first metatarsal and the fifth metatarsal. The entheses included are those of the calcaneal tendon where it attaches to the calcaneus, the attachment sites of the tendons of the *Peroneus longus* muscle and *Tibialis anterior* muscle tendons on the first metatarsal, and the attachment site of the tendon of the *Peroneus brevis* muscle on the fifth metatarsal. These entheses were selected because they are fibrocartilaginous [62, 63] as well as because of the roles these muscles and bones play in foot function (Table 2). Together, the calcaneus, first metatarsal, and fifth metatarsal (connected by the medial longitudinal arch, the lateral longitudinal arch, and the transverse arches) form the tripod over which body mass is distributed while standing [64, 65]. The four muscles listed act to flex the foot up and down and turn it inward and outward, effectively allowing stabilization during standing and walking [66].

**Table 2.** Categorizations of entheses derived from Frowen and Benjamin (1995) and Rufai *et al.* (1995)

Muscle tendon	Insertion of interest	Muscle action	Type of enthesis
Calcaneal tendon (Achilles tendon): attachment for *Plantaris, Gastrocnemius,* and *Soleus* mm	Posterior aspect of calcaneal tuberosity	Foot plantarflexion, flexion of leg at knee	Fibrocartilaginous
Tendon of *Tibialis anterior* m	Inferior/medial aspect of MT1 base	Foot dorsiflexion at ankle and inversion	Fibrocartilaginous
Tendon of *Peroneus longus* m	Inferior tuberosity of MT1 (lateral)	Foot plantarflexion and eversion	Fibrocartilaginous
Tendon of *Peroneus brevis* m	MT5 tuberosity	Foot plantarflexion and eversion	Fibrocartilaginous

Tendon insertions and muscle actions drawn from Stone and Stone (2003).

Entheseal changes were scored and analyzed following the New Coimbra method [67], which was chosen because of its low interobserver error, clear scoring guidelines and inclusion of zonal analysis that accounts for anatomical/functional differences in enthesis structure and subsequent responsiveness to stressors [68]. This method divides fibrocartilaginous entheses into two zones that are scored based on macroscopic observation. Zone 1 is the fibrous part, which runs along the margin of the tendinous origin/insertion, and Zone 2 is the fibrocartilaginous part, which is at the point of attachment itself. At both of these locations, bone formation and erosion are scored. In addition, the following features are scored for Zone 2: textural change, fine porosity, macroporosity and cavitations. Each feature is scored from 1 to 2. In this study, the value ‘0’ was used to indicate the absence of entheseal change. Since the tarsals and metatarsals have not previously been assessed via the New Coimbra method, reference was made to Kelikian [69] to determine the location of Zones 1 and 2.

After scoring each enthesis’ appearance in the eight categories of the New Coimbra method, these scores were combined in two ways for the final analysis: ‘Bone formation score’, which summed Zone 1 and Zone 2 bone formation scores, and ‘Total combined score’, which summed scores in all eight categories. These groupings were informed by the results of multiple correspondence analysis testing, which indicated frequent associations between Zones 1 and 2 bone formation scores. In general, bone formation in both zones was the most common entheseal change observed. Both composite score categories were used to parse differences in entheseal changes related to type of expression: deposition of bone (‘Bone formation score’) and total accumulation of bony changes, both deposition and resorption (‘Total combined score’).

#### Osteoarthritis

Osteoarthritis was recorded for all five metatarsals, calcaneus and talus. These bones were selected because they (with the exception of the talus) interact directly with the substrate and form the tripod for weight distribution when standing. In addition, the joint most frequently affected by osteoarthritis is the first metatarsophalangeal joint, followed by the talocalcaneal joint, talonavicular joint, calcaneocuboid joint and the metatarsocuneiform joints [70, 71]. In this study, osteoarthritis was scored in terms of presence/absence, percentage of articular surface area affected and severity. Severity of osteophytic lesions in the talus, calcaneus and metatarsals was scored for each individual following Buikstra and Ubelaker [72], which score osteoarthritic changes via macroscopic observation. Score categories include degree of lipping (scored from 1 to 4), extent of circumference affected by most severe expression of lipping (scored from 1 to 3), degree of surface porosity (scored from 1 to 3), extent of surface affected by porosity (scored from 1 to 3), degree of eburnation (scored from 1 to 3), and extent of surface affected by eburnation (scored from 1 to 3). In this study, the value ‘0’ was used to indicate the absence of osteoarthritic changes. If multiple articular surfaces of a single bone were affected by osteoarthritic changes, the maximum values of each score were used for analysis.

For ease of comparison between individuals, the scores were combined in three ways: (i) ‘Joint surface score’, which summed scores for degree and extent of surface porosity and eburnation; (ii) ‘Joint periphery score’, which summed scores for degree of lipping and extent of circumference affected by most severe expression of lipping; and (iii) ‘Total combined score’, which summed the joint surface and joint periphery scores. These groupings were used to parse differences in osteoarthritis related to location on the articular surface as well as type of osteoarthritic expression: deposition of bone (‘Joint periphery score’), resorption of bone (‘Joint surface score’) and total accumulation of bony changes, both deposition and resorption (‘Total combined score’). Some studies have suggested that osteophytic lipping along the joint periphery may be linked to early-stage osteoarthritis [73], preceding joint surface changes, while others suggest that lipping is primarily related to aging [52].

#### Biodemographic variables

The biodemographic variables used as covariates for this study were: age at death, sex, stature and body mass index (BMI). Age, sex, stature and weight information was contributed by the donor/donor family and BMI was calculated from this information by the author. Body mass was included as it relates to the loading experienced by the foot bones, joints and muscles. Furthermore, the amount of time spent in sedentary activity increases by BMI in both men and women [16, 18, 22, 74, 75]. Stature was included as it relates to body mass and the overall weight borne by the foot during standing and walking, as well as because it affects gait dynamics. Taller individuals generally exhibit a faster gait speed and longer stride length than shorter individuals [76–78]. In addition to these variables, overall skeletal robusticity may play a role in the expression of osteoarthritis and entheseal changes, especially as greater skeletal robusticity may be an indicator of greater muscle mass (and greater force experienced by entheses). To control for the effects of skeletal robusticity, tibial robusticity was added to statistical analyses as a proxy for overall lower limb robusticity. The tibia was chosen due to its distal placement near the foot. Multiple studies have indicated that the tibia and foot experience similar constraints on bone mass and structure due to their distal placement in the lower limb [79] and that tibial diaphyseal structural properties may be more reflective of locomotive loading than femoral diaphyseal properties [79–82]. Tibial robusticity was calculated as ((maximum diameter at nutrient foramen + mediolateral diameter at nutrient foramen)/bone length) × 100. The tibia selected was from the same side as the tarsals and metatarsals considered, which was usually the right tibia. Tibial robusticity was measured and calculated by the author. Specific information about the lifestyles and physical activity levels of the donors is not known.

### Methods of statistical analysis

All statistical analyses were conducted in R [83].

#### Hypothesis 1

To test Hypothesis 1, ‘Pedal entheseal changes and osteoarthritis are negatively associated with year of birth’, ANCOVA tests were used to determine the direction and magnitude of changes in pedal morphology over time, using year of birth as the explanatory variable and age, sex, stature, BMI, and tibial robusticity as covariates. These tests were used to determine the extent to which temporal trends in pedal morphology can be attributed to temporal trends in biodemographic variables.

#### Hypothesis 2

To test Hypothesis 2, ‘If entheseal changes and osteoarthritis are negatively associated with year of birth, the magnitude of these differences will vary according to sex’, Hypothesis 1 tests were duplicated with the male-only and female-only subsamples. Specifically, ANCOVA tests were used to determine the direction and magnitude of changes in pedal morphology over time for the male- and female-only subsamples, using year of birth as the explanatory variable and age, stature, BMI, and tibial robusticity as covariates.

## RESULTS

### Results of Hypothesis 1 testing

#### Entheseal changes

**Figure 1.**
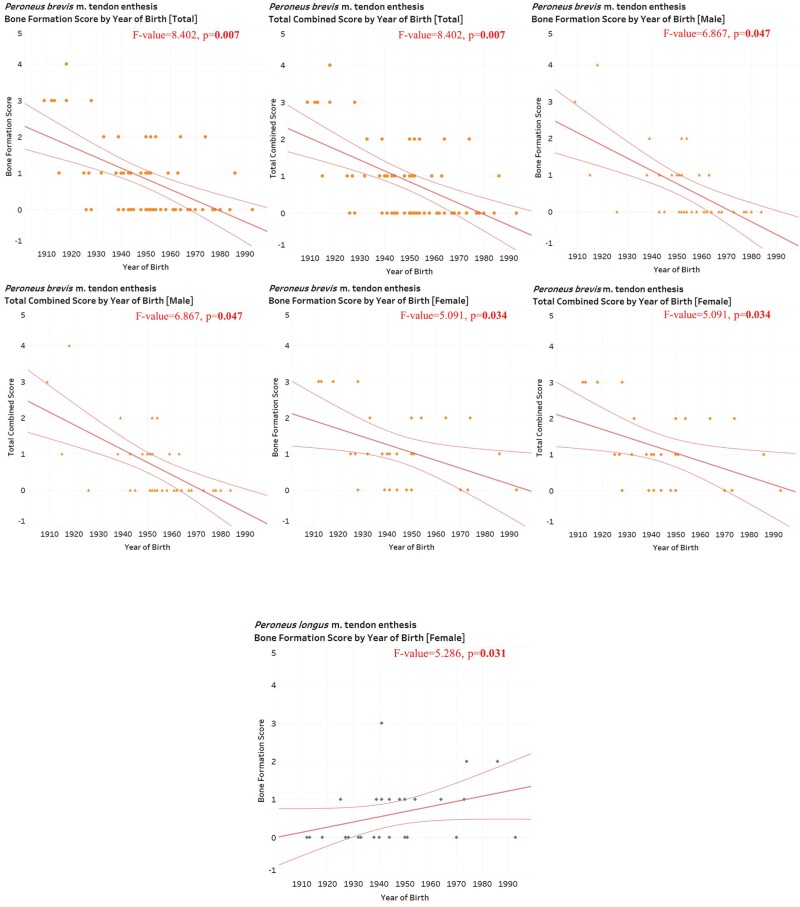
Scatterplots for entheseal changes by year of birth for the total sample (*n* = 71) and male-only (*n* = 43) and female-only (*n* = 28) subsamples. ANCOVA tests indicate that all depicted relationships are statistically significant

ANCOVA tests indicate that there are statistically significant associations between entheseal changes of the *Peroneus brevis* m. tendon enthesis (bone formation score and total combined score) and year of birth in the total sample (Table 3, Fig. 1). This association is negative; that is, individuals born later have less severe entheseal changes than those born earlier (Table 3, Fig. 1). This includes controlling for age at death, so these statistically significant results cannot be attributed solely to the younger age at death of those individuals born later. This result is the only statistically significant result found.

**Table 3.** Results of ANCOVA tests analyzing differences in entheseal scores by year of birth with age, sex, stature, BMI, and tibial robusticity as covariates

				ANCOVA	
Enthesis	Score	Sample	Mean	*F*-value	*P*-value
Calcaneal tendon	Bone formation	Total	1.704	0.083	0.775
		Male	1.512	0.757	0.424
		Female	2.000	0.042	0.840
	Total combined	Total	2.014	0.002	0.969
		Male	1.767	1.589	0.263
		Female	2.393	0.201	0.658
*Peroneus longus* m. tendon	Bone formation	Total	0.437	0.016	0.900
		Male	0.349	4.058	0.100
		Female	0.571	5.286	**0.031**
	Total combined	Total	0.676	0.001	0.973
		Male	0.535	2.305	0.189
		Female	0.893	1.377	0.253
*Tibialis anterior* m. tendon	Bone formation	Total	0.310	0.570	0.456
		Male	0.302	0.150	0.714
		Female	0.321	0.550	0.466
	Total combined	Total	0.451	0.246	0.624
		Male	0.349	0.174	0.694
		Female	0.607	0.164	0.689
*Peroneus brevis* m. tendon	Bone formation	Total	0.803	8.402	**0.007**
		Male	0.581	6.867	**0.047**
		Female	1.143	5.091	**0.034**
	Total combined	Total	0.803	8.402	**0.007**
		Male	0.581	6.867	**0.047**
		Female	1.143	5.091	**0.034**

Statistically significant *P* < 0.05 in bold.

Overall, these results provide partial support for Hypothesis 1.

#### Osteoarthritis

**Figure 2:**
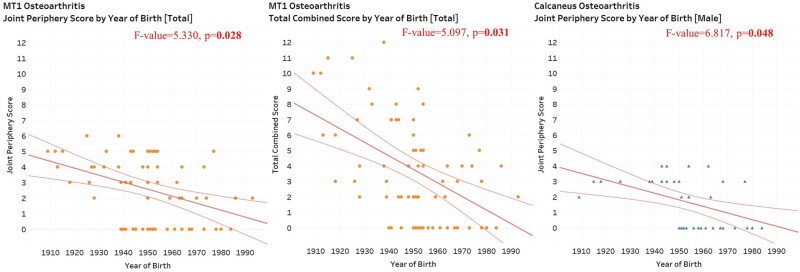
Scatterplots for osteoarthritis by year of birth for the total sample (*n* = 71) and male-only subsample (*n* = 43). ANCOVA tests indicate that all depicted relationships are statistically significant

Temporal trends in osteoarthritis vary in the strength and direction of these relationships by bone. ANCOVA tests indicate that there are statistically significant associations between osteoarthritis of the first metatarsal (joint periphery score and total combined score) and year of birth in the total sample (Table 4, Fig. 2). This association is negative; that is, individuals born later have less severe osteoarthritic changes than those born earlier (Table 4, Fig. 2).

**Table 4.** Results of ANCOVA tests analyzing differences in osteoarthritis scores by year of birth with age, sex, stature, BMI and tibial robusticity as covariates

				ANCOVA	
Bone	Score	Sample	Mean	*F*-value	*P*-value
Calcaneus	Joint surface	Total	0.958	0.084	0.774
		Male	1.023	0.537	0.497
		Female	0.857	0.858	0.364
	Joint periphery	Total	1.676	0.364	0.550
		Male	1.372	6.817	**0.048**
		Female	2.143	0.688	0.416
	Total combined	Total	2.634	0.423	0.520
		Male	2.395	<0.001	0.978
		Female	3.000	1.475	0.237
Talus	Joint surface	Total	0.972	0.369	0.548
		Male	0.977	1.736	0.245
		Female	0.964	0.139	0.713
	Joint periphery	Total	2.200	0.099	0.755
		Male	2.140	0.497	0.512
		Female	2.286	0.069	0.796
	Total combined	Total	3.169	0.068	0.796
		Male	3.116	1.807	0.237
		Female	3.250	0.265	0.612
MT1	Joint surface	Total	1.479	2.729	0.108
		Male	1.070	1.175	0.328
		Female	2.107	1.786	0.195
	Joint periphery	Total	2.309	5.330	**0.028**
		Male	2.025	1.877	0.229
		Female	2.714	3.037	0.095
	Total combined	Total	3.690	5.097	**0.031**
		Male	2.953	1.712	0.248
		Female	4.821	3.314	0.082
MT2	Joint surface	Total	1.662	0.556	0.461
		Male	1.256	0.477	0.521
		Female	2.286	0.777	0.388
	Joint periphery	Total	0.944	1.918	0.176
		Male	0.605	0.801	0.412
		Female	1.464	1.152	0.295
	Total combined	Total	2.606	0.265	0.610
		Male	1.860	0.951	0.374
		Female	3.750	0.025	0.877
MT3	Joint surface	Total	1.507	1.363	0.252
		Male	1.302	<0.001	0.989
		Female	1.821	0.351	0.560
	Joint periphery	Total	0.930	1.435	0.240
		Male	0.558	0.374	0.567
		Female	1.500	0.516	0.480
	Total combined	Total	2.437	0.044	0.835
		Male	1.860	0.302	0.606
		Female	3.321	0.021	0.887
MT4	Joint surface	Total	1.493	0.006	0.938
		Male	1.070	4.230	0.095
		Female	2.143	0.382	0.543
	Joint periphery	Total	0.662	2.609	0.116
		Male	0.558	0.027	0.876
		Female	0.821	3.709	0.067
	Total combined	Total	2.155	0.758	0.391
		Male	1.628	0.801	0.412
		Female	2.964	0.311	0.583
MT5	Joint surface	Total	1.493	0.043	0.837
		Male	1.279	0.510	0.507
		Female	1.821	0.001	0.980
	Joint periphery	Total	0.817	0.741	0.396
		Male	0.651	0.494	0.513
		Female	1.071	2.314	0.142
	Total combined	Total	2.310	0.168	0.684
		Male	1.930	0.015	0.908
		Female	2.893	0.871	0.361

Statistically significant *P* < 0.05 in bold.

This includes controlling for age at death, so these statistically significant results cannot be attributed solely to the younger age at death of those individuals born later. No other significant results were found.

Overall, these results provide partial support for Hypothesis 1.

### Results of Hypothesis 2 testing

#### Entheseal changes

ANCOVA tests indicate that there are statistically significant negative associations between entheseal changes of the *Peroneus brevis* m. tendon enthesis (bone formation score and total combined score) and year of birth in the male-only and female-only subsamples (Table 3, Fig. 1). In addition, ANCOVA tests indicate that there is a statistically significant positive association between *Peroneus longus* m. tendon enthesis (bone formation score) and year of birth in the female-only subsample (Table 3, Fig. 1). This includes controlling for age at death, so these statistically significant results cannot be attributed solely to the younger age at death of those individuals born later. No other significant results were found.

Overall, these results provide partial support for Hypothesis 2.

#### Osteoarthritis

ANCOVA tests indicate that there is a statistically significant negative association between osteoarthritis of the calcaneus (joint periphery score) and year of birth in the male-only subsample (Table 4, Fig. 2). This includes controlling for age at death, so these statistically significant results cannot be attributed solely to the younger age at death of those individuals born later. No other significant results were found in the male-only subsample. ANCOVAs indicate that there are no statistically significant relationships between pedal osteoarthritis and year of birth in the female-only subsample (Table 4).

Overall, these results provide partial support for Hypothesis 2.

## Discussion

### Hypothesis 1

Hypothesis 1, ‘Pedal entheseal changes and osteoarthritis are negatively associated with year of birth’, is partially supported. Specifically, the temporal trends identified in this article support the predictions made in accordance with increased sedentism over time. Entheseal changes and osteoarthritis decrease over time, and these relationships are statistically significant for entheses and bone surfaces of the first (osteoarthritis) and fifth metatarsals (entheseal changes). These temporal trends in entheseal changes (*Peroneus brevis* m. tendon enthesis) and osteoarthritis (first metatarsal joint periphery and total combined scores) are statistically significant when accounting for biodemographic covariates, indicating that these trends cannot be solely attributed to temporal changes in stature, BMI, or tibial robusticity, or to differences in age, sex, stature, BMI, or tibial robusticity among individuals in the sample. These trends are expected considering the changes in activity patterns over the past 100 years, especially the increase in sedentism [20–22, 84, 85]. If individuals are more sedentary, it is expected that they would spend less time on their feet and therefore entheseal changes and osteoarthritis would be less severe.

As mentioned previously, the first metatarsal, particularly the first metatarsophalangeal joint, is the most common site of osteoarthritis in the foot [70, 71]. This joint bears a significant portion of weight during both standing and walking, and therefore changes in physical activity patterns should be expected to have the greatest impact on the first metatarsal. In the present study, these differences were concentrated at the joint periphery rather than the joint surface and were largely related to bone deposition (lipping) rather than resorptive processes. This may be indicative of early-stage osteoarthritis [73]. It is possible that aging also played a role [52], although this statistically significant result accounted for age as a covariate.

The *Peroneus brevis* and *Peroneus longus* mm. both act to plantar flex and evert the foot; however, this role is not shared equally. It is estimated that the *Peroneus brevis* m. ‘provides 63% of total eversion power, as well as assisting in ankle plantar flexion’ [86]. This disproportionate loading of the peroneal muscles may explain why the *Peroneus brevis* m. tendon enthesis should show a decrease in entheseal changes over time compared to the *Peroneus longus* m. tendon enthesis. The *Peroneus brevis* m. also originates much more distally than the other muscles considered in the present study and may therefore more directly reflect loading experienced by the foot.

The results presented here suggest that increased sedentism has affected the skeletal foot. Activity patterns shape the foot by changing the frequency and magnitude of the forces it experiences, from both muscles and joint wearing. Together, both the foot and physical activity patterns embody nationwide cultural shifts related to changing technologies and wealth of the average American.

### Hypothesis 2

Hypothesis 2, ‘If entheseal changes and osteoarthritis are negatively associated with year of birth, the magnitude of these differences will vary according to sex’, is partially supported. The male subsample is the only one to have a statistically significant relationship between osteoarthritis (calcaneus joint periphery score) and year of birth. Relationships between entheseal changes and year of birth are more complicated; both the male- and female-only subsamples have statistically significant relationships between changes of the *Peroneus brevis* m. tendon enthesis and year of birth (that are comparable in terms of significance), while only the female subsample has a statistically significant relationship between changes of the *Peroneus longus* m. tendon enthesis and year of birth. There is a difference, then, both in magnitude of temporal trends as well as the entheses/bone surfaces that are affected in the male- versus female-only subsamples. In addition, the female subsample is unique in being the only subsample to exhibit a positive relationship between pedal health and year of birth; specifically, this is the relationship between entheseal changes of the *Peroneus longus* m. tendon enthesis (bone formation score) and year of birth. These results suggest that the loading on some bones, joints, and muscles has increased over time for women, in contrast to the male-only and total sample trends.

Since these relationships between pedal health variables and sex remain statistically significant when accounting for biodemographic covariates, something else must be driving this sexual dimorphism. It is possible that this is related to hormonal differences between men and women, as these are known to influence bone health. For example, Villotte and Knüsel [40] draw on clinical literature [87, 88] to suggest that women may be more susceptible to entheseal changes due to ovarian hormones such as estradiol and relaxin that ‘promote hyper-laxity and increase the risk of intrinsic mechanical lesions’. This sexual dimorphism in entheseal changes could also be attributed to the aforementioned differences in physical activity between males and females during the last century. During the past 100 years, men and women’s activity patterns changed in two main ways: (i) women’s activity patterns changed as they took on occupations outside of the home, and (ii) men began engaging in sedentary behaviors to a greater degree than women. Still, even while working outside the home, there has been the expectation for women to remain the primary caretaker and homemaker [58, 59]. This may result, then, in women engaging in overall more physical activity (and more time spent on their feet) due to the combination of occupational and homemaking responsibilities.

The increase in pedal entheseal changes in the female-only subsample may exemplify gendered cultural practices that have become physically embodied, particularly in regard to behaviors involving plantar flexion and eversion. Interestingly, this statistically significant positive relationship is only seen for the bone formation score, not the total combined score, potentially indicating that this positive trend is specifically embodied via bone formation at the *Peroneus longus* m. tendon enthesis. Perhaps, like has been suggested for osteoarthritis [73], bone formation is a precursor to degenerative entheseal changes and indicates early-stage changes to the *Peroneus longus* m. tendon enthesis. Considered with the decrease in changes to the *Peroneus brevis* m. enthesis, this may be indicative of plantar flexion and eversion motions being transferred from the *Peroneus brevis* m. to the *Peroneus longus* m., rather than these actions being absolutely reduced as in the male-only subsample. Bavdek *et al.* [89] found that the *Peroneus longus* m. exhibits higher mean electromyography amplitudes when walking on an incline ramp with elevated lateral foot than the *Peroneus brevis* m. Therefore, frequent use of a more everted foot position may contribute to such a transfer of load-sharing between the peroneal muscles. This may also be related to ankle instability: with fatigue, women exhibit decreased ankle plantar flexion/dorsiflexion stiffness and increased *Peroneus longus* m. reflex amplitude compared to men [90]. The same increase in reflex amplitude is not seen for the *Peroneus brevis* m [90].

Unfortunately, these conclusions are limited due to small sample sizes and sex imbalances in the study group, especially in the 46–55 year age range (Table 1). The sexually dimorphic patterns in entheseal changes established in this study require further exploration with larger samples.

### Potential impacts on quality of life

Although sedentism is often detrimental to bodily health, the decrease in entheseal changes and osteoarthritis presented here might be considered a positive development as they suggest lower stress overall on the modern human foot. Ultimately, the net gain of these temporal changes is dependent on the magnitude of temporal changes; entheseal changes can be painful, especially when accompanied by inflammation [91–95], so a reduction in entheseal changes would be positive. A decrease in entheseal changes may also reflect less stress on entheses and muscles themselves, reducing the chance of overuse injuries, but a decrease in muscle mass below a certain level may put the foot and ankle at risk of injury due to lack of joint support.

Overall, a decrease in foot osteoarthritis would be a net positive for humans. Foot osteoarthritis has many negative effects on quality of life: pain, swelling, a reduced range of motion, reduced foot and leg muscle strength, difficulty walking and the development of secondary conditions like hallux limitus or hallux rigidus [70, 96, 97]. These effects, in turn, lead to impaired balance and an increased risk of falls [98–100], an increased risk of developing knee pain and knee osteoarthritis [101, 102], and a reduction in mental health, including increased risk of anxiety and depression [103]. Foot pain is disabling and may lead individuals to significantly alter their daily life activities to avoid standing and walking [54, 104–106]. In this way, foot osteoarthritis may lead to increased participation in sedentary behaviors, which protect against the development of more severe foot osteoarthritis but may have other negative consequences like lower limb muscle atrophy [96, 107]. Interestingly, the findings of this present study conflict with those concerning secular trends in knee osteoarthritis over the same period. For example, Wallace and colleagues [108] found an increase in knee osteoarthritis from the mid-20th century to the present. These authors suggest that this increase in knee osteoarthritis may be attributed to thinner joint cartilage and weaker muscles surrounding the joints in less physically active individuals, which predisposes their joints to damage.

In considering foot health in our modern environment, some authors have suggested that the foot may be currently undergoing microevolutionary changes due to changing cultural stressors [65, 109]. An increase in anatomical variants in the foot may also reflect a microevolutionary trend due to relaxed selection [109]. It is possible that the decrease in osteoarthritis and entheseal changes found in this study may be explained by a relaxation of cultural stressors due to increasing time spent sedentary. Although it has been hypothesized that the mismatch between our current environment and our evolutionary environment has contributed to poor health in modern humans, this may be one aspect of human health that has improved due to environmental changes (increased sedentism).

Ultimately, these findings should be considered in conjunction with other studies of secular trends in foot morphology and health, such as Kryst *et al.* [110]. This study assessed foot length and breadth in children and adolescents in Kraków, Poland in 2010 and 2020 and found that foot length decreased over time while foot breadth increased. The authors suggest that these trends may be related to cultural environmental factors, including secular increases in body weight and increases in sedentary behavior, but do not consider how these morphological changes may affect foot function and quality of life. Previous studies have found that wider feet contribute to a more even distribution of peak pressures across the foot [111], perhaps explaining the decreases in entheseal changes and osteoarthritis found in the present study. Considering that pathological changes to the skeleton often imply chronic issues that take time to develop in the bone, the decrease in entheseal changes and osteoarthritis found here may, in conjunction with secular increases in soft tissue foot maladies, indicate that foot problems are not as severe or chronic as they have been in the past. Instead of being visible in the bone, they are only visible at the level of the soft tissue (and are perhaps medically addressed before progressing further).

### Limitations

These conclusions are currently limited by small sample sizes, but future studies will expand this analysis of entheseal changes in the foot skeleton. This study’s conclusions may also be limited by the nature of this sample. The majority of individuals in the Bass Collection are from Tennessee and the Southeastern USA [112], and this particular sample was comprised of only white individuals. Although the expression of entheseal changes should be generally applicable to the modern human person regardless of ancestry or region, it is possible that these results are instead representative of ancestry or region-specific entheseal expression. Comparison of these results with other skeletal collections will be useful in determining whether the temporal trends documented in the Bass Collection are unique to this collection, this time period and this region of the USA.

It is possible that the secular trends in entheseal changes and osteoarthritis established here may additionally be explained by trends in footwear that have occurred over the past 100 years. In general, the past 100 years have been characterized by trends toward wider shoes with more cushioned soles [113–116] that may decrease the forces experienced by the bones, joints and muscles of the foot, thereby leading to the decrease in entheseal changes and osteoarthritis seen here. For example, Landry *et al*. [117] found that conventional shoes with artificial support features are overprotective and cause underutilization of many foot muscles. including the peroneal muscles. This explanation should be further explored in donated skeletal collections with documented footwear.

## CONCLUSIONS AND IMPLICATIONS

This study addressed the question, ‘To what degree has sedentism impacted pedal health over the last 100 years in the United States?’ The results present the following conclusions:

1) Over the past 100 years, an increase in sedentism has contributed to a decrease in pedal entheseal changes and osteoarthritis over time in some regions of the foot.
2) Temporal trends in pedal entheseal changes and osteoarthritis vary by sex. In one instance (*Peroneus brevis* m. enthesis bone formation score), pedal entheseal changes have increased in females.

This research has highlighted differences in the relative influence of both biodemographic and cultural variables on the health of the pedal skeleton. Although biodemographic variables clearly contribute to variation in pedal health, the decrease in entheseal changes and osteoarthritis over time remains statistically significant even when they are included as covariates, suggesting that the secular trend observed here is also related to changes in life habits in the period studied, such as the increase in sedentism. This study supports the hypothesis that the individuals who donated their bodies to the Bass Collection physically incorporated gendered differences in cultural practice via their tarsals and metatarsals. In particular, these sex-specific trends in pedal variables are likely related to the activity patterns characteristic of each sex over the last century. These conclusions are currently limited by small sample sizes, but future studies will expand this analysis of sexual dimorphism and gendered cultural practice.

This project furthers our understanding of how our modern, culturally constructed environment shapes the morphology of our skeleton [118]. Changes in the frequency and severity of foot osteoarthritis and entheseal changes may reflect embodiment of nation-wide changes in the US economy, increased suburbanization and the increased affordability and accessibility of indoor entertainment, as these changes impact occupations that are available, the average household income, and societal expectations and valuations regarding leisure time. These societal changes, in turn, influence an individual’s activity patterns at work and at home. Physical activity (or lack thereof) then directly impacts the foot skeleton as it determines the constraint and loading experienced by the foot during growth, development and adulthood. These aspects of our culturally constructed environment are, evolutionarily speaking, relatively recent changes to the suite of environmental stressors that our feet experience. This research therefore highlights a potential microevolutionary trend that may be resulting from the mismatch in our evolutionary and modern environments. Conversely, understanding the links between skeletal changes of the foot and health in the present can also help us identify and interpret foot paleopathology related to lifestyle in the archaeological and paleoanthropological record. This study provides a path to exploring such topics in future studies utilizing documented skeletal collections or interpreting human remains in archaeological contexts.
